# Decoding the autotetraploid origin of the stress-tolerant wild beet *Beta corolliflora*

**DOI:** 10.1186/s12864-026-13276-8

**Published:** 2026-09-26

**Authors:** Katharina Sielemann, Nicola Schmidt, Jonas Guzik, Natalie Kalina, Boas Pucker, Prisca Viehöver, Sarah Breitenbach, Tony Heitkam, Daniela Holtgräwe

**Affiliations:** 1 Genetics and Genomics of Plants, Center for Biotechnology (CeBiTec) & Faculty of Biology, Bielefeld University, Bielefeld, 33615 Germany; 2 Graduate School DILS, Bielefeld Institute for Bioinformatics Infrastructure (BIBI), Bielefeld University, Bielefeld, 33615 Germany; 3 Molecular Botany, Institute of Biology I, RWTH Aachen University, Aachen, 52074 Germany; 4 Plant Biotechnology and Bioinformatics, Institute for Cellular and Molecular Botany (IZMB), University of Bonn, Kirschallee 1, Bonn, 53115 Germany; 5 Cell and Molecular Biology of Plants, Faculty of Biology, Technische Universität Dresden, Dresden, 01069 Germany

**Keywords:** Sugar beet, *Beta corolliflora*, *Beta lomatogona*, *Beta macrorhiza*, *Patellifolia procumbens*, Crop wild relatives, Polyploidy, Genome sequencing, Parental relationship, *K*-mer

## Abstract

**Background:**

Most crop plants, including sugar beet (*Beta vulgaris* subsp. *vulgaris*), suffer from domestication bottlenecks and low genetic diversity caused by extensive selection for few traits. However, crop wild relatives (CWRs) harbour useful traits relevant for crop improvement, including enhanced adaptation to biotic and abiotic stresses. Especially polyploids are interesting from an evolutionary perspective as the genome undergoes reorganisation after the polyploidisation event. Through neo- and subfunctionalisation, novel gene functions emerge, which enable plants to cope with changing environments and extreme/harsh conditions. To introduce such resilience traits into breeding material, CWRs have already been identified as an important source for sustainable breeding. For beets, the section *Corollinae* contains the tetraploid species *Beta corolliflora* (2n = 4x = 36) that is believed to harbour salt and frost tolerances as well as a wealth of pathogen resistances. The number of beneficial traits of *B. corolliflora* is increased compared to those of the known diploids in this section (all 2n = 2x = 18). Nevertheless, neither the parental relationships of *B. corolliflora* have been resolved, nor are genomic resources available to steer sustainable, genomics-informed breeding.

**Results:**

To benefit from the resources offered by (polyploid) beet wild relatives, we generated and evaluated genome resources and assemblies for four different sugar beet wild relatives - *Beta corolliflora* (tetraploid), *Beta lomatogona* (diploid), *Beta macrorhiza* (diploid), and as an outgroup *Patellifolia procumbens* (diploid). We combined cytogenetic, k-mer-, and gene-based approaches that present strong evidence for the parental relationship of the *B. corolliflora* wild beet as an autotetraploid emerging from *B. macrorhiza*. Together with the publicly available genome sequences of two additional wild beets, we identified genomic regions absent from the cultivated beet, providing a sequence database harbouring traits relevant for future breeding endeavours.

**Conclusion:**

The provided evidence for the evolutionary history of wild beets, especially *Beta corolliflora*`s derivation from the diploid *B. macrorhiza*, resolves long-standing questions and highlights how genomic data can clarify crop evolution and support breeding.

**Supplementary Information:**

The online version contains supplementary material available at https://doi.org/10.1186/s12864-026-13276-8.

## Background

### Sugar beet, crop wild relatives and the potential for breeding

The crop plant sugar beet (*Beta vulgaris* subsp. *vulgaris*) is of high economic relevance contributing to approximately 20% of the global sugar production [1]. To increase sugar production, early breeding focused mainly on yield. The domestication process introduced a strong genetic bottleneck resulting in diminished diversity available to breeders [2, 3]. Other important traits, like resistances to biotic and abiotic stresses, were initially neglected but gain more and more relevance in the face of climate change [4]. It was already shown that some sea beets and some wild beets contain agronomically important traits that were lost during domestication [1]. Examples for such traits include salt and nematode tolerances [2, 5, 6, 7]. However, other crop wild relatives (CWRs) of sugar beet might harbour even more potential in terms of traits which can be incorporated to allow more sustainable beet cultivation [2]. To this end, we sequenced and assembled the genome sequences of four different wild beets, namely *Beta corolliflora*, *Beta lomatogona*, *Beta macrorhiza*, and *Patellifolia procumbens*.

### Resolving the origin of the polyploid wild beet *Beta corolliflora*

Polyploid organisms are evolutionarily interesting as genomes often undergo reorganisation after the polyploidisation event, and genes gain room for neo- or subfunctionalisation. Novel functions can emerge, enabling the plant to better adapt to changing environments and stressful conditions [8, 9, 10, 11]. This is not only true for allopolyploids, where the genomes of two different species are combined, but also for autopolyploids that evolve from one diploid parent. Despite the extensive niche overlaps of progenitor and descendant, these ploidy increases can stabilise heterosis, resulting for example in higher adaptability to stress [11, 12]. Regarding beets and wild beets, the section *Corollinae* harbours a range of higher polyploids. Among them, the most well-known is the tetraploid *Beta corolliflora* (2n = 4x = 36, 1 C genome size = 2027 Mb) [13]. *B. corolliflora* is, just like most beets, an outcrosser [14] and is also described as an apomictic plant, i.e. performing asexual reproduction via seeds [15]. It harbours a wide range of beneficial traits, including salt and frost tolerance as well as various resistances against pathogens [2]. Yet, the type and origin of its polyploidy remain unclear. Having a diploid chromosome configuration of 2n = 2x = 18, *B. lomatogona* (1 C genome size = 929 Mb) and *B. macrorhiza* (1 C genome size = 936–946 Mb) [13] were considered as potential parents. These species are the only known diploids of the section *Corollinae* that show a geographical distribution overlap with *B. corolliflora* [16]. *Beta corolliflora* is therefore considered to be either an allotetraploid resulting from hybridization of *B. lomatogona* and *B. macrorhiza*, or an autotetraploid resulting from a whole genome duplication of only one of those two species (or closely related to extinct relatives of one of those two species) [17, 18]. A comprehensive sequencing dataset can serve as basis to trace the origin of *B. corolliflora*’s tetraploidy and may provide important insights into the polyploidisation event.

In this study, we present evidence for the tetraploid origin of *B. corolliflora* by generating short- and long-read datasets as well as genome sequence assemblies for four different sugar beet wild relatives: *B. corolliflora* (4x), *B. lomatogona* (2x), *B. macrorhiza* (2x), and as an outgroup *P. procumbens* (2x). These newly available beet genomic resources, together with the genome sequence of the cultivated sugar beet reference KWS2320 (assembly version KWS2320ONT v1.0; *B. vulgaris* subsp. *vulgaris*) [5], sea beet (*B. vulgaris* subsp. *maritima* WB42) [19], and *B. patula* [19], were used to gain first insights into the beet pangenome by employing cytogenetic, *k-*mer-, and gene-based methods to get evidence for the parental relationships of the tetraploid wild beet *B. corolliflora.*

## Methods

### Plant material

The Leibniz Institute of Plant Genetics and Crop Plant Research Gatersleben (IPK), Germany, provided seeds for *B. corolliflora* (BETA 408), *B. lomatogona* (BETA 674), *B. macrorhiza* (BETA 830), and *P. procumbens* (BETA 419). The material was transferred under the regulations of the standard material transfer agreement (SMTA) of the International Treaty. All plants were grown under long day conditions (16 h light, 8 h dark) at 18–24 °C in the greenhouse.

### DNA extraction, sequencing and *de novo* assembly

All assemblies were generated from DNA extracted from a single plant.

For *B. macrorhiza*, Illumina short reads were generated, as only a low amount of plant material was available, which was not sufficient for extracting high molecular DNA suitable for long read sequencing. High molecular weight DNA was extracted using a previously described CTAB-based method [20]. DNA extraction for short-read sequencing as well as the Illumina sequencing itself was performed as previously described [16]. In total, 138 Gbp read data were generated for *B. macrorhiza* (Additional file 4). For the draft assembly process, these reads were trimmed using Trimmomatic (v0.39) [21] as described before [16] and the quality was assessed using FastQC (v0.11.9) [22]. All trimmed reads were subjected to DiscovarDeNovo (v52488) (run with default parameters and 10 threads; https://www.broadinstitute.org/software/discovar/blog) [23] for *de novo* genome assembly after converting the FASTQ files to unmapped BAM files with picard tools (v2.5.0) (http://broadinstitute.github.io/picard/). Contigs with a length below 500 bp were discarded. ‘Decontamination’ of the assembly, i.e. discarding sequences with matches to a ’black list’ (bacterial or fungal genome sequences and highly overrepresented sequences), was performed as described previously [20]. In this case, sequences with perfect matches against the genome sequences of *Arabidopsis thaliana*, *Vitis vinifera*, and Trifoliate yam (*Dioscorea dumetorum*) were discarded as well, as these species had been sequenced in the laboratory during the same months as the beet genomes. The completeness of the final genome sequence assemblies (Additional file 5) was assessed using BUSCO (v5.2.2) [24] (embryophyta_odb10 dataset, -m genome, -c 10).

Long reads were generated for the species *B. corolliflora*, *B. lomatogona*, and *P. procumbens* using Oxford Nanopore Technologies (ONT*)*. Quality control for genomic DNA was performed by agarose gel electrophoresis, NanoDrop measurement, and Qubit analysis [20]. The short read eliminator kit (Circulomics) was used to remove short DNA fragments. Library preparation was conducted based on the SQK-LSK109 protocol (ONT). Sequencing was performed on a GridION using R9.4.1 flow cells as described previously [20] (Additional file 4). For *B. corolliflora* and *B. lomatogona*, basecalling was performed using Guppy (v3.2) (https://nanoporetech.com/). Super high accuracy basecalling (v6) was available for read data from *P. procumbens*. The N50 read lengths for *B. corolliflora*, *B. lomatogona*, and *P. procumbens* are approx. 19.0 kbp, 15.9 kbp, and 25.7 kbp, respectively. A *de novo* assembly for each species was generated with Canu (v.1.8; for *P. procumbens*: v2.2) (parameters, excluding memory/threads: useGrid = 1, saveReads=true, corMhapFilterThreshold = 0.0000000002, ovlMerThreshold = 500, corMhapOptions=--threshold 0.80, --num-hashes 512, --num-min-matches 3, --ordered-sketch-size 1000, --ordered-kmer-size 14, --min-olap-length 2000, --repeat-idf-scale 50) [25]. Polishing of all ONT assemblies was performed with racon [26], followed by two rounds of medaka (https://github.com/nanoporetech/medaka) and three rounds of pilon [27] as described previously [20]. Contigs below 100 kbp were discarded. ‘Decontamination’ of the assembly, i.e. discarding sequences with matches to a ’black list’, was performed as described previously [20]. The completeness of the final genome sequence assemblies (Additional file 5) was again assessed using BUSCO (v5.2.2) [24] (embryophyta_odb10 dataset, -m genome, -c 10).

### Gene prediction and functional annotation

Prior to gene prediction, softmasking of the repeats in all genome assembly sequences was performed. A *de novo* repeat library was constructed with RepeatModeler (v2.0) [28] including the LTR discovery pipeline. The RepBase library for each species together with the species-specific RepeatModeler library were used as input to RepeatMasker (v4.1.1) [29].

The BRAKER2 pipeline [30, 31, 32, 33, 34, 35, 36, 37, 38] was used for gene prediction. Protein evidence, derived from OrthoDB protein sequences [39] formatted with ProtHint [32], as well as full-length sugar beet mRNA sequences from RefBeet-1.0 and BeetSet 2.0 [40, 41] were integrated as hints. The full-length mRNA sequences were aligned to the respective genome sequence assembly using BLAT [42] (parameters: -fine; -q = rna). The alignments were filtered (filterPSL.pl; --best, --minCover = 80, --minId = 92), sorted by sequence names and begin coordinates, and then transformed into GFF format (blat2hints.pl). Both hint files, derived from RefBeet-1.0 and RefBeet1.5, were compared by alignment positions to discard the respective RefBeet-1.0 mRNA in case of an overlap with a RefBeet-1.5 mRNA. The alignments were merged to obtain the final hints file. The actual gene prediction was performed with BRAKER2 in the ‘etpmode’. Several scripts were used to reformat the resulting annotation file (fix_gtf_ids.py, gtf2gff.pl, augustus_to_GFF3_adapName.pl). Predicted genes encoding a polypeptide shorter than 50 amino acids were removed.

Each gene was named according to a species abbreviation composed of the first letter of the genus name and the first letter of the species name (e.g. *P. procumbens*: Pp). The contig name was added after an underscore and then followed by the gene number (sorted by assembly coordinates). The last part of the gene name is composed of four-letter codes - either based on reciprocal best hits (RBHs) identified by BLASTn against RefBeet genes, or by a new four-letter combination if no match to an existing RefBeet gene model was observed in any annotation version.

All genes were functionally annotated by InterProScan (v5.52) [43], SwissProt, BLASTX [44] and RBH-BLAST using published RefBeet annotations. The functional annotation files are available as part of this study (https://doi.org/10.4119/unibi/2966932).

### Computational methods to resolve polyploid relationships

An overview of the read (Additional file 4) and assembly datasets (Additional file 5), used for the different approaches to resolve the ancestral relationships of *B. corolliflora*, is provided. Assembly statistics were calculated with QUAST (v. 5.2.0) [45].

### *K*-mer approaches to resolve polyploid relationships

#### Technical background

To efficiently search specific *k*-mers in a given *k*-mer set, we generated a data structure based on the first six positions of the *k-*mers. For this, we grouped all *k*-mers starting with the same six ‘letters’, i.e., bases, into buckets, i.e., all *k*-mers in a bucket have the same prefix. This corresponds to building a prefix tree (static trie) over the prefixes. As the prefix tree already encodes the prefixes, only the suffixes in the buckets have to be saved in the form of sorted arrays. One can test for the occurrence of a *k*-mer *m* by first traversing the tree along the prefix of *m* until a bucket is reached. If this traversal fails, *m* is not present in the set. If a bucket is reached successfully, a binary search in the bucket is performed. The application ‘*k*-mer operator’ (SBTTrio application; https://github.com/ksielemann/relationships_tetraploid_wild_beet), which enables such searches, was written in Java.

#### Generating unique *k*-mers

To extract unique *k*-mers from a sequence, a sliding window of length *k* is moved over the sequence, i.e., all substrings of length *k* are captured and stored. To get all canonical *k*-mers, the reverse complement of each *k*-mer is calculated and the lexicographically smaller one is selected as the canonical *k*-mer. Each *k-*mer and its reverse complement are therefore represented by a single form. All canonical *k-*mers are then inserted into a hash table using the hash function MurmurHash3 [46]. An open addressing hash table with a size that is always a power of 2 was used together with a quadratic probing function ($\:\frac{i\left(i\mathrm{+}1\right)}{2}$) [47].

The developed method can also be used to generate random sets of canonical *k*-mers. To achieve optimal time and space complexity at sampling (without replacement) random *k*-mers, a specific algorithm was used (sparse Fisher-Yates shuffle) [48].

### *K*-mer set operations

Similarities and differences between the species-specific *k*-mer sets were assessed using various set operations. As input for the *k*-mer set operations method, we first used normalised Illumina read datasets of the potential ancestor species to achieve a comparable set size. Normalisation was performed with bbnorm [49] and the parameters *k* = 21, a target depth of 20 and a minimum threshold of 3 to discard likely erroneous reads. For the descendant species, the complete set of *k*-mers based on the read datasets was used. In addition, the set operations were performed using sequence assemblies as input.

For each investigated species dataset, the set of distinct canonical *k*-mers (*k* = 13, 21, 31) was computed using the *k*-mer counting algorithm KMC3 (v3.2.1) [50]. Based on the species-specific *k*-mer sets, multiple subsets were generated using different set operations. This includes the subset of *k*-mers present in all species (*B. corolliflora*, *B. macrorhiza*, and *B. lomatogona*), shared among two species as well as the subset of *k*-mers shared by two species, but not present in the third investigated species. These set operations were performed with KMC tools (v3.2.1) [50].

### Generalised trio binning

Trio binning is used to separate reads into two haplotype-specific sets to generate phased assemblies [51]. This approach was adapted here to assess the ancestral contributions to tetraploid *B. corolliflora*. First, the *k*-mer set (*k* = 21) for the potential ancestor species was calculated with KMC3 using either assemblies or high-quality, normalised (as described above) short reads as input. Then, the set of exclusive *k*-mers for each of the potential ancestor species was calculated with KMC tools (i.e. the set of *k*-mers present in one species, but not in the other). For the descendant species, a long-read dataset was used. For each read in this dataset, the number of unique canonical *k*-mers this read shares with the exclusive *k*-mer set of one of the ancestor candidate species was counted using the ‘*k*-mer operator’ described above. This number is then divided by the number of unique canonical *k*-mers of the read to get the ‘*k*-mer share’ for each potential ancestor. The average share of exclusive *k*-mers assigned to the potential ancestors was calculated over all reads. Only reads, for which at least half of the average *k*-mer share was assigned to the potential ancestors, are considered (the other reads contain too few *k*-mers exclusive to either one of the potential ancestor species). The goal of this filtering is to exclude reads where the overall signal is too weak to be interpreted reliably. All remaining reads were then classified into four different classes (Additional file 6): if the number of exclusive *k*-mers of a specific read is 3x higher for ancestor (parent) A than for ancestor (parent) B, the read was assigned to ancestor A (i) (beige) and (ii) vice versa (orange-red) (Additional file 6). The read was classified as ‘chimeric’ (iii) if $\:\frac{k\mathrm{-}mershareparentA}{k\mathrm{-}mershareparentB}\mathrm{-}\frac{1}{2}\vee\:0.05$ (orange). If none of the three conditions above applied, the read was ‘unclassified’ (IV) (grey). Generalised trio binning (SBTTrio application; https://github.com/ksielemann/relationships_tetraploid_wild_beet) was performed for the trio of interest (*B. corolliflora*, *B. lomatogona*, *B. macrorhiza*) as well as for other control trios to validate the approach (Table 1). *B. oleracea* (696 Mb) contributes a higher sequence content to allotetraploid *B. napus* in comparison to *B. rapa* (529 Mb) [52]. For this reason, we normalised the results for this genome size difference.

**Table 1:** Results of the generalised trio binning approach.

Analysis	Input	Descendantspecies	Ancestor A	Ancestor B	Unclassified	Chimeric
Test case	Reads	Bcor	Blom: 0.031	Bmrh: 0.519	0.173	0.029
Test case	Assemblies	Bcor	Blom: 0.115	Bmrh: 0.4	0.257	0.05
Control allo	Reads	Bnap	Bole: 0.291	Brap: 0.245	0.061	0.009
Control ‘auto’	Assemblies	Bvul	Bpat: 0.047	Bmar: 0.356	0.307	0.066

For each trio, the type of analysis, the type of input datasets used to generate the *k*-mer sets, the name of the descendant species as well as the proportion of reads assigned to each of the four categories (Ancestor A/B, Unclassified, Chimeric) are provided

*Abbreviations:Bcor*  B. corolliflora, *Blom*  B. lomatogona,* Bmrh * B. macrorhiza, *Bnap*  B. napus, *Bole*  B. oleracea, *Brap*  B. rapa, *Bvul * B. vulgaris subsp. vulgaris, *Bpat*  B. patula, *Bmar*  B. vulgaris subsp. maritima, *allo*  allopolyploidy, auto  autopolyploidy

### *K*-mer fingerprinting

The *k*-mer fingerprinting approach introduces randomisation and is motivated by Fofanov et al. [53] which suggests that small *k*-mer sets can be used to distinguish different organisms with high probability. The randomisation is also motivated by the prospect of minimising the impact of errors and therefore having a closer reflection of the real similarity when using the average over multiple random canonical *k*-mer sets. In general, this approach is similar to *k*-mer sketching.

To select a suitable *k*, the percentage of distinct canonical *k*-mers that are present in the dataset of each species was computed for a range of *k* (14–20). In accordance with Fofanov et al. [53] a *k* was selected for which 5%-50% of all possible unique canonical *k*-mers were present in all datasets. This ensured that the different species datasets can be distinguished and that erroneous *k*-mers of low-quality reads do not impact the results. In general, the choice of *k* is a trade-off between a clear signal and computational intensity. In contrast to the previously described *k*-mer-based approaches, for which *k* = 21 was well suitable, here, *k* = 15 was selected based on the criterion by Fofanov et al. [53], and KMC3 was used to generate *k*-mer sets. The ‘*k*-mer operator’ was used to build indices for all investigated datasets and to generate 100,000 random sets of size 10,000 based on the whole set of all theoretically possible 15-mers. For each random set, the fingerprint, i.e. overlap with the species-specific *k*-mer set, was calculated. Additionally, the fingerprint intersection, i.e. the overlap of the fingerprint of the descendant species with the respective fingerprint of each investigated potential ancestor species, was computed.

*K*-mer fingerprinting was performed on assembly sequences for *B. corolliflora* as descendant species and *B. lomatogona*, *B. macrorhiza*, *B. patula*, *B. vulgaris* subsp. *maritima*, *B. vulgaris* subsp. *vulgaris*, and *P. procumbens* as candidate ancestor species.

### Mapping approach to resolve polyploid relationships

#### Synthetic read mapping

As input, synthetic reads were generated from the sequence assembly of the descendant species (*B. corolliflora*). These synthetic reads were extracted by splitting each contig into equal length fragments starting from the beginning of the contig. The synthetic reads were then mapped simultaneously against the sequence assemblies of the two potential ancestor species (*B. lomatogona* and *B. macrorhiza*) using minimap2 within the corresponding Python wrapper mappy (v2.24) [54]. The reads were then assigned to four categories either (i) mapping to both potential ancestors, (ii) mapping exclusively to ancestor A, (iii) exclusively to ancestor B, or (iv) not mapping to either of the ancestor candidates. A synthetic read length of 5 kbp, 10 kbp, and 20 kbp was selected and only primary mappings with a sequence identity of at least 60% (to allow sequences with assembly errors and with higher divergence between species to be included in the analysis) were considered. For comparability between the *B. lomatogona* long read-based and the *B. macrorhiza* short read assembly, in a second approach, the *B. lomatogona* assembly sequence was shredded into 5 kbp (= smaller than the N50 of the *B. macrorhiza* assembly) chunks prior to the mapping procedure.

#### Gene-based approach to resolve polyploid relationships

Phylogenetic distances of BUSCO gene sequences were calculated between tetraploid *B. corolliflora*, the potential ancestors, *B. vulgaris* subsp. *vulgaris*, *P. procumbens* and three related outgroup long-read genome sequence assemblies of the Caryophyllales (*Simmondsia chinensis* (jojoba; GCA_018398585.1), *Spinacia oleracea* (spinach; GCF_002007265.1), and *Amaranthus hypochondriacus* (amaranth; GCA_000753965.2)). For all eight species, BUSCO (v5.2.2) [24] (embryophyta_odb10 dataset) was run in genome mode. Afterwards, suitable BUSCO genes were extracted. The final set of single copy (can be duplicated in the tetraploid *B. corolliflora*), complete BUSCO genes present in all six genome sequences comprised 140 genes. A multiple FASTA file with amino acid sequences was constructed for each gene and served as input for sequence alignment with MAFFT v7.299b (L-INS-I method; --adjustdirection) [55]. The alignments were trimmed with trimAl (v1.4.rev22) [56] to achieve 100% occupancy, which means that only single nucleotide variants (SNVs) were considered for the phylogenetic distance whereas insertions/deletions (InDels), possibly derived from assembly or gene structure annotation errors, were not considered. Single gene trees were constructed using FastTree (v2.1.11) [57] The phylogenetic distance of each ancestral gene to the closest related *B. corolliflora* gene was assessed using the DendroPy library [58]. A Mann-Whitney-U test, implemented in the SciPy package [59], was calculated.

#### Chromosome preparation and fluorescent in situ hybridisation

Mitotic chromosomes were prepared from young meristematic leaves of *B. corolliflora* (BETA 408), *B. lomatogona* (BETA 674) and *B. macrorhiza* (BETA 830) as described previously [60, 61]. Probes for the satellite DNAs pRN1 [62]. GenBank accession number Z69354.1) and BlSat1 [63] were labelled by PCR in the presence of biotin-16-dUTP (Roche Diagnostics) detected by streptavidin-Cy3 (Sigma–Aldrich) or digoxigenin-11-dUTP (Jena Bioscience) detected by antidigoxigenin-fluorescein isothiocyanate (FITC; Roche Diagnostics). The 18 S rDNA probe was labelled with DY415-dUTP (Dyomics). All probe nucleotide sequences are listed in the Additional file 7. Chromosomes were counterstained with DAPI (4′,6′-diamidino-2-phenylindole; Böhringer, Mannheim) and mounted in antifade solution (CitiFluor; Agar Scientific, Stansted). The hybridization procedure as well as the image acquisition were performed as described previously [60, 64]. The hybridization stringency was 79%.

## Results

### Genome sequencing data of wild beets

In order to investigate the ancestral relationships of tetraploid *B. corolliflora* by the application of different *k*-mer- and gene-based approaches, we generated short- and long-read sequencing data. Further, to test the different approaches and to provide comprehensive genomic resources, three long read-based assemblies (*B. corolliflora*: BcorONT v1.0, *B. lomatogona*: BlomONT v1.0, and *P. procumbens*: PproONT v1.0) and a short read-based assembly (*B. macrorhiza*: Bmrh v1.0) of wild beet species were generated (Additional file 1). The largest genome sequence assembly was constructed for the tetraploid *B. corolliflora* with a total size of approximately 1.96 Gbp, similar to the expected genome size determined by flow cytometry (Additional file 1) [13]. The genome sequence assemblies of the diploid species *B. lomatogona* and *P. procumbens* have a comparable approximate total assembly size of 1 Gbp with 1500 contigs each. The final assembly for *B. macrorhiza* comprises 218,216 contigs with a cumulative size of 736 Mbp and does not reach the expected genome size of 936–946 Mbp [13]. Here, limited access to leaf material restricted the DNA amounts, resulting in an Illumina-only assembly. All newly generated assemblies exceed the size of the KWS2320 sugar beet reference genome sequences Refbeet-1.2, RefBeet-1.5, KWS2320ONT v1.0, and RefBeet-3.0 [40, 65, 5, 66]. In general, all long read-based assemblies show solid completeness with BUSCO percentages above 90% (Additional file 1). The number of non-single copy (at least duplicated) BUSCOs is substantially higher for the tetraploid species (72.9%), with most of the complete BUSCOs being triplicated in BcorONT v1.0. The assemblies of section *Corollinae* species show a slightly higher GC content (average of BcorONT v1.0, BlomONT v1.0, and Bmrh v1.0: 36.58%) compared to section *Beta* (35.74% in KWS2320ONT v1.0; 35.8% in EL10) [5, 67]. The repeat content is similarly high in all genome sequences ranging from 64.23% in Bmrh v1.0 to 71.73% in BcorONT v1.0, but generally higher in species with larger genomes [13].

### Resolving ancestral relationships of tetraploid *B. corolliflora*

**Fig. 1:**
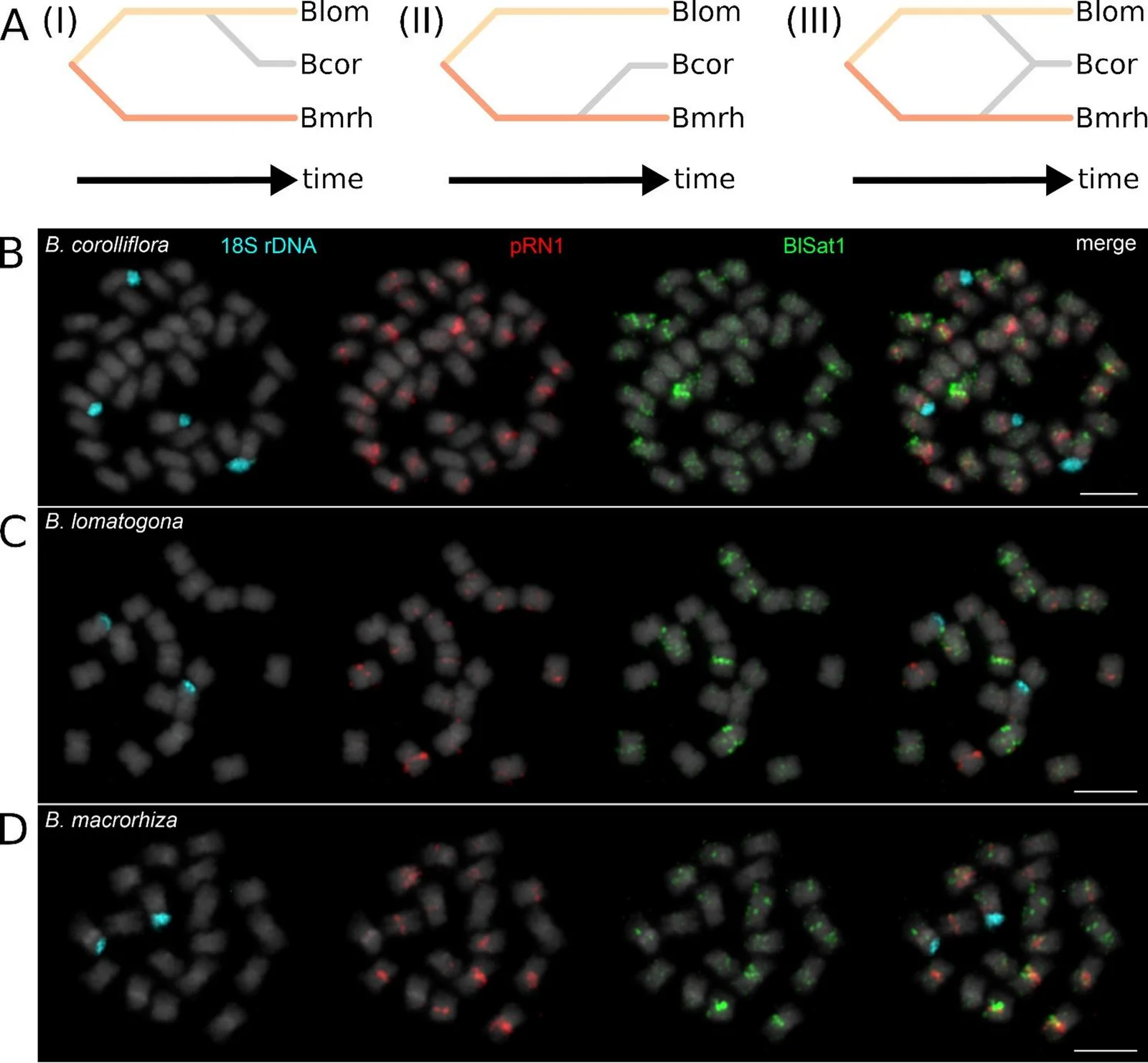
Possible ancestorrelationships of tetraploid *B. corolliflora* (Bcor) and their support by cytogenetics. **A **Hypothesis (I) shows *B. corolliflora* as autotetraploid species with *B. lomatogona* (Blom) being the single ancestor species. Hypothesis (II) shows *B. macrorhiza* (Bmrh) as a single ancestorof autotetraploid *B. corolliflora*, whereas hypothesis (III) considers both ancestorss contributing to allopolyploid *B. corolliflora*. **B**-**D**: Chromosomal landmarks along mitotic chromosomes of *B. corolliflora*, *B. lomatogona* and *B. macrorhiza* point towards hypothesis (II), but are not sufficient to unequivocally deduce the ancestral relationships. Mitotic chromosomes of the wild beets *B. corolliflora* (**A**), *B. lomatogona* (**B**), and *B. macrorhiza* (**C**) were hybridised with probes marking the 18 S rDNA gene (with DY415; blue signals) and the satellite DNAs pRN1 (with streptavidin-Cy5; red signals), BlSat1 (with antidigoxygenin-FITC; green signals). The 18 S rDNA is a widely used cytogenetic mark, usually flagging one chromosome pair in beets [68, 19]. The chromosomes were counterstained with DAPI (grey). See Additional file 2 for signal counts and interpretation

To demonstrate the power of the wild beet genome sequencing data, we addressed the question of the ancestral relationships of tetraploid *B. corolliflora*. For this, we consider three different hypotheses that target the emergence from *B. lomatogona* and *B. macrorhiza* (Fig. 1A). These hypotheses are: autotetraploidy originating from either diploid *B. lomatogona* (I) or *B. macrorhiza* (II) and allopolyploidy originating from hybridization of both diploid species (III).

### Cytogenetics

First, we studied how the three genomes compare on a chromosomal level. Logically, after tetraploidization, the chromosomes from the diploid(s) should be found again in the chromosomal set of the tetraploid. Here, we show a cytogenetics approach using three probes derived from tandemly repeated DNA sequences, that hybridize across all three *Corollinae* species (Fig. 1B-D). We then evaluate the number and strength of each produced signal to test the cytogenetic support for each of the three hypotheses brought forward (Additional file 2). The rationale behind this cytogenetic test is the following: (I) an autotetraploid origin of *B. corolliflora* with *B. lomatogona* as single ancestor species would show cytogenetic signals that correspond to the doubled signals of *B. lomatogona*, both in number and signal strength. (II) Similarly, an autotetraploid origin with *B. macrorhiza* as single ancestor species would show cytogenetic signals that correspond to the doubled signals of *B. macrorhiza*, both in number and signal strength. (III) An allotetraploid origin with both diploids as ancestors would be most supported by additive signals of both diploids (signal number and strength), see the detailed signal counts in Additional file 2.

The 18 S rDNA probe, a widely used cytogenetic mark (Fig. 1B-D, blue), distinctly labels four chromosomes in the tetraploid (Fig. 1B) and two chromosomes in the diploids (Fig. 1C and D), not discriminating between the three hypotheses. Therefore, as the remaining two probes, we chose tandemly repeated satellite DNAs that occur solely in wild beets of the *Corollinae* [13] and have potential to inform about genetic differences between the wild beet species. For beetSat10-pRN1, we observe hybridization on 32 chromosomes, with many major and moderate signals in *B. corolliflora* (Fig. 1B red; signal counts in Additional file 2). Similarly, beetSat8-BlSat1 hybridizes to all chromosomes with varying intensity (Fig. 1B green; signal counts in Additional file 2). Then, we comparatively hybridized these probes to *B. lomatogona* and *B. macrorhiza* chromosomes (Fig. 1C and D; Additional file 2, A) to deduce expected signal counts for each hypothesis and to calculate how each count varies from the expectation (Additional file 2, B-D). As a result, we find least differences between the observed and the expected signal counts for hypothesis (II). Hence, we conclude most cytogenetic support for *B. corolliflora*’s emergence through autotetraploidization of *B. macrorhiza* (hypothesis II) but also acknowledge the limitations of the cytogenetic analysis.

### Computational approaches

To verify and confirm the tetraploid ancestry of *B. corolliflora* using genome sequence data, we deployed five different computational approaches. Some of these approaches are based directly on Illumina reads as input data (read-based approaches) and are therefore not dependent on assembly quality parameters.

### K-mer composition of the investigated genome sequences

**Fig. 2:**
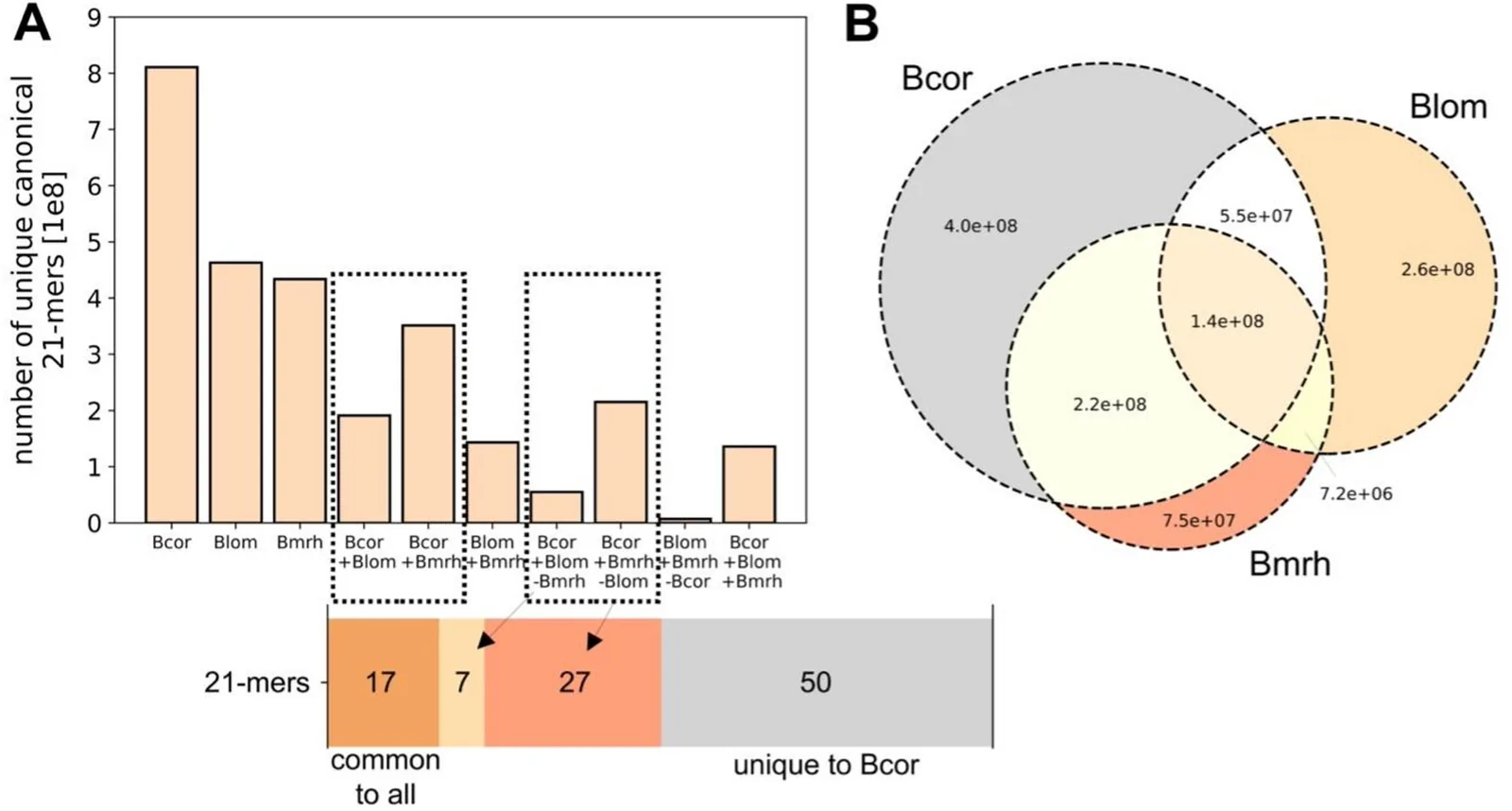
Results of the *k*-mer set operations for each tested hypothesis. **A **Size (number of unique canonical 21-mers) of all investigated sets. The bars within the left black box represent intersections between the *B. corolliflora* (Bcor) specie and its putative ancestor species (Blom or Bmrh). The box on the right side represents the 21-mer set sizes including 21-mers present in only one candidate ancestor species but not in the other. The horizontal bar plot below summarises the composition of the *B. corolliflora* 21-mer set. **B **Venn diagram for the read-based 21-mer sets of *B. corolliflora (Bcor)*, *B. lomatogona (Blom)*, and *B. macrorhiza (Bmrh)*

First, we looked at the *k*-mer composition of the genome sequences. The similarity of the genome sequences of two species reflects the distance of their relationship. In turn, the similarity of two sequences is reflected by the similarity of their *k*-mer sets. Essentially, the set of *k*-mers of a sequence equals a compact representation of that sequence. Additionally, comparing *k*-mer sets is assumed to be more robust than directly comparing sequences considering assembly errors, e.g. at repetitive regions. The horizontal bar plot (Fig. 2A) summarises the composition of the *B. corolliflora* 21-mer set. *Beta corolliflora* shares 27% of its *k*-mers exclusively with *B. macrorhiza* and 7% exclusively with *B. lomatogona*.

The overlap of the read-based (derived from the sequencing reads) *k*-mer sets of *B. corolliflora*, *B. lomatogona*, and *B. macrorhiza* was visualised in a Venn diagram (Fig. 2B). The *k*-mer set of *B. macrorhiza* has a substantially higher intersection/overlap with the *B. corolliflora k*-mer set (3.5e8; 81% of the whole *B. macrorhiza* set) compared to the *B. lomatogona k*-mer set (1.9e8; 41% of the whole *B. lomatogona* set).

### Assigning reads to ancestor candidates based on similarity

In a second approach, ‘generalised trio binning’ was performed: We adapted the established trio binning approach [51] used to assign reads from a descendant into haplotype-specific sets to resolve parental or more generally phylogenetic relationships. The rationale behind this approach is that the number of reads assigned to one of the ancestor candidates reflects its relationship to the descendant relative to the other potential ancestor’s relationship to the descendant species. For both, the assembly- and the read-based trio, including *B. corolliflora*, *B. lomatogona*, and *B. macrorhiza*, the proportion of reads assigned to *B. macrorhiza* (0.4 and 0.519, respectively) is substantially higher than the proportion of reads assigned to *B. lomatogona* (0.115 and 0.31) (Table 1).

As a control trio for a well-known allopolyploid species complex, datasets of *Brassica oleracea* and *Brassica rapa* as known ancestors of *Brassica napus* were analysed. A similar proportion of *B. napus* reads was assigned to both ancestral species (0.291 and 0.245). Here, *B. napus* is considered both genomes combined, with 696 Mb from *B. oleracea* (contributing 56.8% to the *B. napus* genome) + 529 Mb from *B. rapa* (contributes 43.2% to the *B. napus* genome). Normalising the results for these genome size differences results in a proportion of reads assigned to *B. oleracea* and *B. rapa*, of 0.512 and 0.567, respectively. The similar amount of *B. napus* reads assigned to both ancestors shows that the method leads to the expected results.

As another control, *B. vulgaris* subsp. *maritima* as known progenitor of *B. vulgaris* subsp. *vulgaris* was used together with *B. patula* which is not considered to be a progenitor of *B. vulgaris* subsp. *vulgaris*. These species are no polyploids, however, the progenitor-descendant relationship of these species is known, which enables further validation of our approach. For this trio, a clear signal towards *B. vulgaris* subsp. *maritima* is visible (0.356) whereas a proportion of 0.047 of the reads are assigned to *B. patula*.

### Using random subsets of *k*-mer sets to determine similarity

In a third *k*-mer based approach, *k*-mer fingerprints for numerous random sets were computed. As already described for the *k*-mer set operations approach, the more closely related two species are, the more similarity is expected between the respective *k*-mer sets. For this, we randomly generated 100,000 sets of 10,000 15-mers each based on the whole set of all theoretically possible 15-mers. For each set of 10,000 15-mers we then calculated the overlap with the species-specific *k*-mer set – the so-called fingerprint.

The average absolute fingerprint sizes, i.e. the number of 15-mers overlapping between the random set of 10,000 15-mers and the species-specific *k*-mer set, are shown in Table 2. *Beta corolliflora* has the largest average fingerprint size (4,358). *Beta patula*, *B. vulgaris* subsp. *maritima*, and *B. vulgaris* subsp. *vulgaris* show a similar average fingerprint size in the range of 3,061 to 3,088. *Beta macrorhiza* shows the largest fingerprint intersection (3093; 92.7%), i.e. the overlap between the *B. macrorhiza* set with the set of the descendant species *B. corolliflora* (Table 2). This value is substantially higher than the one of the other putative diploid ancestor *B. lomatogona* (2822; 77.1%). Considering the diploids from the section *Beta*, *B. patula*, *B. vulgaris* subsp. *maritima*, and *B. vulgaris* subsp. *vulgaris* have similar fingerprint intersection sizes (2235–2251, 73.0%). The smallest fingerprint intersection size is observed for the wild beet representative from the sister genus *Patellifolia*, *P. procumbens* (2179; 66.4%).

**Table 2:** Results of the *k*-mer fingerprinting approach

Species	Average fingerprint size (= average number of 15-mers of the species also occurring in the random 15-mer sets of size 10,000)	Absolute average fingerprint intersection size with B. corolliflora (= average number of 15-mers in the first column/average fingerprint size, which also occur within the 15-mer set of B. corolliflora)	Relative average fingerprint intersection size with B. corolliflora [%]
*B. corolliflora*	4358 ± 50	-	-
*B. lomatogona*	3658 ± 48	2822 ± 45	77.1
*B. macrorhiza*	3335 ± 47	3093 ± 46	92.7
*B. patula*	3076 ± 46	2245 ± 42	73.0
*B. vulgaris* subsp. *maritima*	3088 ± 46	2251 ± 42	73.0
*B. vulgaris* subsp. *vulgaris*	3061 ± 46	2235 ± 42	73.0
*P. procumbens*	3284 ± 47	2179 ± 41	66.4

2nd column: Average fingerprint sizes of all random sets of size 10,000 for all investigated species. 3rd and 4th column: Absolute and relative (relative with respect to the ancestor candidate) average fingerprint intersection sizes of all ancestor candidates. In addition to the average values, the standard deviations are shown

### Synthetic reads reflect the relationship between species

The fourth approach applied relied on cross-species mapping of ‘synthetic reads’, i.e. sequence fragments of the assembled contigs of equal length. It is expected that the closer two species are related, the higher their sequence similarity. Therefore, it is expected to find more sequence regions of the assembly of species A in the assembly of species B, the closer these species are related. Based on these assumptions, a mapping approach was developed to present evidence for the ancestor relationships of tetraploid *B. corolliflora*. This mapping approach to resolve the ancestor relationships of *B. corolliflora* directly compares the two candidate ancestors. Additional file 3 shows the percentage of synthetic *B. corolliflora* reads that mapped exclusively to *B. lomatogona*, exclusively to *B. macrorhiza* or to both species with a sequence identity of at least 60%. For all considered synthetic read lengths (5 kbp, 10 kbp, and 20 kbp), the percentage of reads that map to both potential ancestors is below 1%. With shorter read length, the percentage of reads mapping only to *B. macrorhiza* increases whereas the percentage of reads mapping only to *B. lomatogona* decreases. For 5 kbp reads, more than twice as many successfully mapped reads map exclusively to *B. macrorhiza* v1.0 (68% versus 31% for BlomONT v1.0). When mapping 5 kbp reads against the reference consisting of *B. lomatogona* shredded into 5 kbp chunks and the short read assembly of *B. macrorhiza*, the results are almost identical to the ones using the full-length *B. lomatogona* assembly as reference.

### Similarity of orthologous genes

**Fig. 3:**
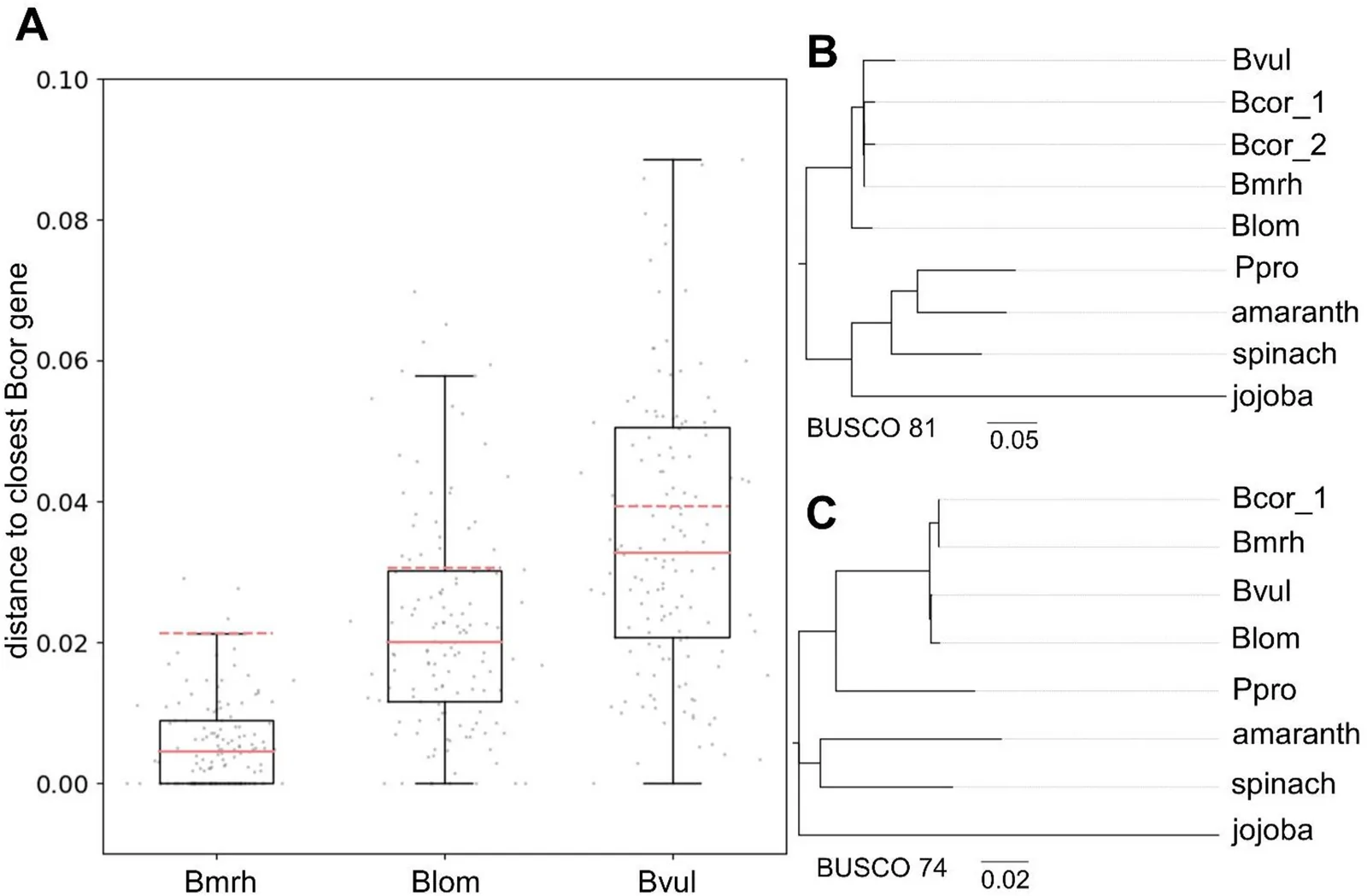
Results of the gene-based approach to determine the ancestral relationships of *B. corolliflora*. **A** Phylogenetic distance of all ‘parental’ genes to the respective closest related *B. corolliflora* gene. The mean is shown as a dashed orange line whereas the median is represented by a solid orange line. **B**, **C** Phylogenetic ML trees for two selected BUSCO genes. Bcor_1 and Bcor_2 represent two different copies of the same gene in *B. corolliflora* (duplicated BUSCO). Spinach (*S. oleracea*), amaranth (*A. hypochondriacus*), and jojoba (*S. chinensis*) were used as outgroup species. Abbreviations: Bcor = *B. corolliflora*, Blom = *B. lomatogona*, Bmrh = *B. macrorhiza*, Bvul = *B. vulgaris* subsp. *vulgaris*, Ppro = *P. procumbens*

In a fifth approach, that is based on gene sequences (not on *k*-mers), the similarity of orthologous genes was used as a measure to assess the putative ancestors of tetraploid *B. corolliflora*. A large basis of SNVs in single-copy BUSCO genes was employed to calculate phylogenetic distances of the gene sequences of the potential ancestors to the respective gene sequence of *B. corolliflora*. For this, 140 single BUSCO gene phylogenies were computed. The phylogenetic distance of the *B. macrorhiza* genes to the respective closest related *B. corolliflora* gene (mean approx. 0.0213) is significantly smaller when compared to *B. lomatogona* (mean approx. 0.0305) (U-test; *p* ≈ 5e-24) (Fig. 3A). This means that *B. macrorhiza* genes are substantially more often found in a common phylogenetic unit together with the respective *B. corolliflora* gene, whereas *B. lomatogona* genes are often found on a separate branch in the phylogenetic tree. Two examples on BUSCO genes for this observation are given in Fig. 3B3C).

To gain more insight, phylogenetic distances in trees in which the *B. macrorhiza* gene is not clustered with the closest *B. corolliflora* gene were further investigated. Branch lengths in such trees are particularly small and *B. corolliflora*, *B. lomatogona*, and *B. macrorhiza* sequences of the respective genes are hardly distinguishable with phylogenetic distances of e.g., < 0.0086 (average distance between two sequences in this tree: 0.104197).

### Absent regions in sugar beet but present in the wild beets – The tetraploid *B. corolliflora* genome is especially rich in candidate gene regions

As especially polyploid CWRs might harbour properties/traits not present in the cultivated beet [2], the newly generated sequence resources, including tetraploid *B. corolliflora*, were used to identify regions not present in the KWS2320 sugar beet breeding material. Here, we compare the tetraploid *B. corolliflora* to a range of diploids across the two beet genera *Beta* and *Patellifolia* and show the usefulness of including a polyploid species into a pangenome dataset. Absent regions in the cultivated sugar beet KWS2320, but present in the CWRs, were defined as follows. If the region is not present in sugar beet, (almost) no reads of sugar beet should map to the corresponding region in any of the crop wild relatives. Based on this rationale, ‘zero coverage regions’ (ZCR) were extracted. Through a functional annotation that was generated for BcorONT v1.0, BlomONT v1.0, Bmrh v1.0, and PproONT v1.0 (https://doi.org/10.4119/unibi/2966932), the set of identified CWR genes was investigated for specific disease resistance genes (R-genes), genes conferring tolerances, and genes related with response to bacteria, viruses, or fungi.

For the investigated CWRs, 4.0% (*B. maritima*) to 10.2% (*B. patula*) of the genome sequence assemblies were found to be ZCRs and therefore to be absent and/or not present in sugar beet genotype KWS2320 (Additional file 8). For the *B. corolliflora*, *B. lomatogona* and *B. macrorhiza* assembly, very similar values (4.7% to 5,0%) for ZCRs were found. Most of the genes in the ZCRs lack a functional annotation, but several genes were found to be related to plant defence against pathogens or to response to abiotic stress like heat, frost and salinity. As an example, 39 different disease resistance proteins and putative disease resistance proteins were collectively identified for all four species in the functional annotations. Only 16 of them were present in the annotation of at least two species, the remaining 23 were unique to one of the species. The annotation for *B. corolliflora* contained all of the five (putative) R-genes *RGA1-5*, *B. lomatogona* and *B. macrorhiza RGA1-4*, and *P. procumbens RGA3*. *B. corolliflora* contained most unique resistance genes (22) compared to the other species investigated. Genes that are unique to *B. corolliflora* were e.g. *Resistance to Pseudomonas syringae pv. Maculicola 1 (RPM1)*, *Probable disease resistance protein (DAR5)*,* Resistance Silenced Gene 2 (RSG2)*, and the putative late blight resistance protein homolog *R1B-19* (*Solanum demissum*).

## Discussion

To investigate the ancestor relationships of *B. corolliflora*, a sequencing read dataset as well as draft genome sequence for four different wild beets (*B. corolliflora* (2n = 4x), *B. lomatogona* (2n = 2x), *B. macrorhiza* (2n = 2x), and *P. procumbens* (2n = 2x)) were generated. Most analyses presented here can be based on *k*-mers derived from sequencing reads only. Through comparison to the read-based results and the gene-based approach, it was shown that our genome sequence assemblies are suitable to investigate ancestor relationships. Published genome sequences of *B. patula* and *B. vulgaris* subsp. *maritima* [19] as well as the long read-based genome sequence of cultivated sugar beet (KWS2320ONT v1.0) [5] were integrated to get evidence for the ancestor relationships of the tetraploid beet *B. corolliflora*.

### *B. macrorhiza* as single ancestor of autotetraploid *B. corolliflora*

We combined multi-colour cytogenetics with five computational approaches to elucidate the type of tetraploidy in *B. corolliflora* and its ancestry. Using all six approaches, we can now confidently exclude *B. lomatogona* as ancestor, and we find comprehensive evidence of an emergence as autotetraploid from *B. macrorhiza*. Alternatively, as an option that we cannot distinguish from the autotetraploid scenario, *B. corolliflora* might be an allotetraploid derived from two different but closely related *B. macrorhiza* genotypes.

To define the diploid ancestry of a polyploid is a question that is and has been commonly addressed using cytogenetics [69, 70, 71]. Here, as the ancestor genomes are closely related with relatively limited variation amongst cytogenetic probes, the question is answered only with difficulty and not conclusively. Still, our cytogenetic analysis retained most support for *B. corolliflora*’s autotetraploidy emerging from *B. macrorhiza*, and was finally verified by computational genomics.

To convincingly resolve the question of *B. corolliflora*’s tetraploidy, we leveraged five data-driven genomics approaches. The advantage of the three included *k*-mer approaches is that these approaches do not rely on a reference genome sequence and are not dependent on e.g. the identification of homology through computationally expensive (whole genome) alignment approaches [72, 73].

For all *k*-mer based approaches, it is important to take the genome size of the potential ancestors into account. The *k*-mer set size is dependent on the genome size and also on the size of the assembly, since the probability of a *k*-mer occurring just by chance grows with increasing genome sequence size. For polyploids, the haploid genome size might be more relevant. An additional copy of a genome, e.g. diploid vs. autotetraploid, does not increase the *k*-mer set size linearly. However, rearrangements, transposable element expansions, and particularly small mutations occurring after the polyploidisation/hybridisation increase the potential for additional *k*-mers. In addition to the biological genome size, the completeness and therefore the quality of the assembly has similar effects on the *k*-mer set size. Even though the assembly quality for *B. macrorhiza* is lower compared to the quality of the long read assemblies, there is a striking signal towards *B. macrorhiza* for all approaches. Further, the number of unique *k*-mers is dependent on the heterozygosity of a genome (higher heterozygosity equals more unique *k*-mers). Such differences in the number of unique *k-*mers might have to be considered in the investigation of other ancestor relationships. In our case, the number of unique *k-*mers is similar for both ancestor candidates (see Fig. 2A, second and third bar).

The composition of the *B. corolliflora* 21-mer set (Fig. 2A) shows a higher overlap with the *B. macrorhiza* set than with the *B. lomatogona* set, indicating a closer relationship of *B. corolliflora* and *B. macrorhiza*. A higher *k*-mer set similarity implies a higher sequence similarity and therefore closer phylogenetic relationship. As visualised in the Venn diagram (Fig. 2B), both the absolute and the relative intersection sizes of *B. corolliflora* and *B. macrorhiza* are greater than those of *B. corolliflora* and *B. lomatogona*. Especially considering the fragmented assembly of *B. macrorhiza*, this supports the robustness of the *k*-mer set operations.

For trio binning, if both investigated species were the actual ancestor species of the descendant, it was expected that approximately the same number of reads would be assigned to both supposed ancestors. If only one of the candidate species was the ancestor, substantially more reads should be assigned to the designated species. This number depends on the phylogenetic relationship of the second candidate to the descendant species. Multiple factors, however, may lead to a divergence from these expectations: unequal genome sizes of both ancestors lead to a higher expected number of reads assigned to the species with the larger genome. Bias during the process of sequencing may also lead to an uneven distribution of reads [74]. Read quality, coverage, allelic variation, and complex genomic regions all affect the effectivity of trio binning and can explain why a rate of 100% of assigned reads cannot be achieved. Further, rearrangements and sequence differences originating during the species’ evolution, especially of the genome of the descendant species may distort read distribution and *k*-mer content. The number of sequence differences depend on how long ago the polyploidization event occurred and since rearrangements and extended genome divergence are regularly observed in polyploid species [10] the trio binning approach is mainly aimed at resolving the ancestor relationships of young hybrid species.

Two ‘control trios’ were selected to validate the generalised trio binning approach and show that the method leads to the expected results. As allotetraploid control, the *B. napus*, *B. oleracea*, and *B. rapa* trio was used [75]. A similar number of reads was assigned to both known ancestors of *B. napus*. The slightly higher number of reads for *B. oleracea* can be explained by the larger 21-mer set size. The second control trio comprises the sea beet as known progenitor of sugar beet [1, 76] as well as *B. patula*, not a progenitor of sugar beet. More than seven times more reads are assigned to the sea beet compared to *B. patula*, which confirms the close relation of sea beet and sugar beet.

Regardless of using assemblies or reads as input for a trio of interest, substantially more reads are assigned to *B. macrorhiza*. Again, an advantage of this approach is that it does not rely on assembled data, even though it is possible to use assembled data as input. Using assemblies as input, *B. macrorhiza* obtains about four times more reads, whereas using reads as input, about 17 times more reads are assigned to *B. macrorhiza* than to *B. lomatogona*. These results indicate that *B. macrorhiza* might be the single ancestor of autoploid *B. corolliflora*. The difference in the results when using assemblies versus reads as input can be explained by the *k*-mer set sizes derived from the assemblies of *B. lomatogona* (3.5e8) and *B. macrorhiza* (2.8e8). The assembly-based *k*-mer set for *B. macrorhiza* is smaller, which can be explained by the fragmented short read assembly in which *k*-mers exclusive to unassembled regions might be missing. Using reads as input, it can be assumed that the normalised read datasets reflect the true *k*-mer sets well. Indeed, the difference in the size of the exclusive *k*-mer sets when using reads is smaller (3.2e8 for *B. lomatogona* and 2.9e8 for *B. macrorhiza*).

The idea of the *k*-mer fingerprinting approach is similar to the *k*-mer set operations method, however, there are two major differences: (i) the randomisation introduced in the fingerprinting method can reduce the impact of errors when taking the average over a sufficiently large number of random sets [53] and (ii) this approach allows to compare more than two ancestor candidates simultaneously. The average fingerprint sizes are mainly related to genome size and sequence diversity (Table 2). *B. corolliflora* shows the largest average fingerprint size since the genome sequence is the largest among the investigated organisms. Considering the relative fingerprint intersection sizes, the results reflect the phylogenetic relationships of the species [16]. *Patellifolia procumbens* has the highest phylogenetic distance to *B. corolliflora* among the investigated organisms and shows the smallest relative fingerprint intersection size. The fingerprint intersection sizes of all other investigated species also directly reflect the phylogenetic distances. The substantial difference in average relative fingerprint intersection size between *B. lomatogona* (77.1%) and *B. macrorhiza* (92.7%) suggests that *B. macrorhiza* is more closely related to *B. corolliflora* and presumably the single ancestor species. For the *k*-mer fingerprinting approach, an additional comparison with *B. nana* and *B. intermedia*, two additional species of the section *Corollinae*, would have been interesting, however, the required data was not available.

The synthetic read mapping approach reflects the similarity between sequence sections (synthetic reads) of *B. corolliflora* and the assembly sequences of the potential ancestor species. An advantage of this approach is the equal coverage distribution of the descendant species’ synthetic reads close to one. Therefore, specific regions are not substantially over- or underrepresented and the results are not biased by such sequences. Further, such synthetic, contig-based reads likely contain fewer errors than the actual sequencing reads the contigs are based on. The decrease in the percentage of reads which exclusively map to *B. macrorhiza* with increasing synthetic read length (Additional file 3), can be explained by the high fragmentation of the *B. macrorhiza* assembly. For synthetic reads of 5 kbp length, more than twice as many reads map exclusively to *B. macrorhiza* compared to *B. lomatogona*. This indicates a higher sequence similarity and thus also a closer relationship between *B. macrorhiza* and *B. corolliflora* as opposed to *B. lomatogona* and *B. corolliflora*.

The gene-based approach was developed to assess the sequence similarity of conserved BUSCO genes [24] between descendant and potential ancestor species. These investigated sequences are more similar between *B. macrorhiza* and *B. corolliflora* as shown by the clusters in the phylogenetic tree separate from the respective *B. lomatogona* gene sequence.

**Fig. 4:**
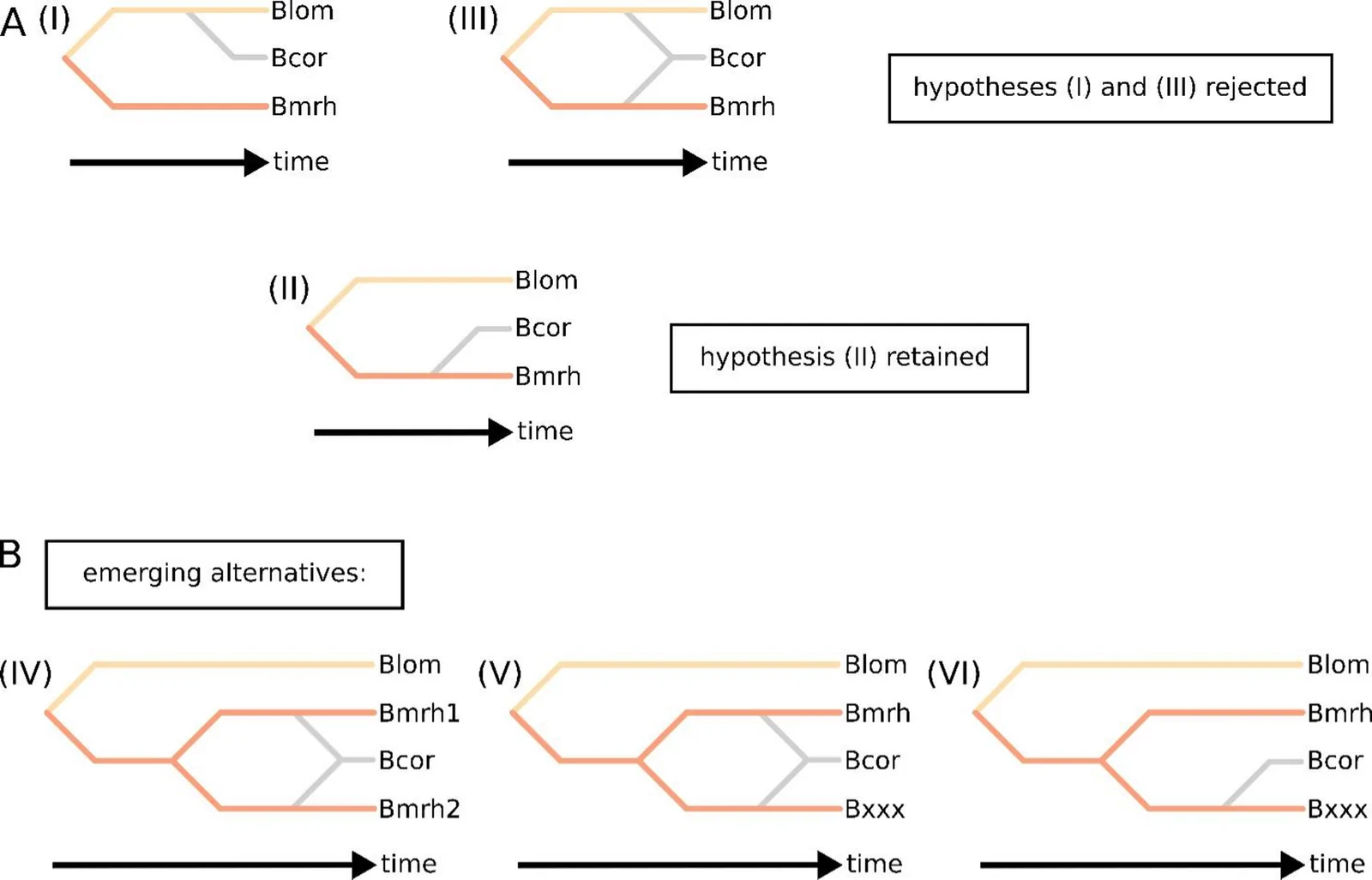
Amended hypotheses regarding the origin of tetraploid *B. corolliflora*. From the initial three hypothesis (**A** I-III), two hypotheses were disproved (I and III). *B. corolliflora* (Bcor) might be an autotetraploid species with Bmrh as a single ancestor (hypothesis II). This possibility is sharpened by the emergence of three new hypotheses (**B** IV-VI), in which *B. corolliflora* either originated from the hybridisation of two different *B. macrorhiza* cytotypes (IV), of *B. macrorhiza* with an unknown, possibly extinct *Beta* species (Bxxx) closely related to *B. macrorhiza*, or from the autopolyploidisation from this unknown *Beta* species (VI). However, these hypotheses cannot be tested with the available data as the existence of Bxxx is unknown

Combining all our results, cytogenetics and the five computational approaches, a clear pattern towards resolving *B. corolliflora*’s ancestry emerges: All computational approaches show a clear signal, while the cytogenetics approach shows no decisive evidence, but also no contradictory signal. In summary, our analyses indicate that *B. lomatogona* is not likely to be in an ancestor of the tetraploid wild beet but show a clear signal towards *B. macrorhiza* being the ancestorspecies of *B. corolliflora*, which therefore would be most likely an autotetraploid species. However, we cannot exclude the possibility that the real ancestor of *B. corolliflora* is an unknown and possibly already extinct species very closely related to *B. macrorhiza*, or that *B. corolliflora* originated from a hybridisation event of such an unknown species with *B. macrorhiza* (Fig. 4). Further, allo- and autopolyploidy are considered to reside along a ‘spectrum’ [77]: (i) highly diverse subgenomes from a single species can lead to the formation of a more polymorphic autopolyploid as compared to an allopolyploid species derived from two less diverged species. (ii) Homoeologous exchanges can contribute to the formation of a partially autopolyploid species from an initial allopolyploid. This means that different regions of the genome appear to be allopolyploid whereas other regions appear to be autopolyploid. (iii) Directional selection of genes, which favours one of the ancestor genomes, may cause homeologs to ‘appear’ autopolyploid [77] even though the species is originally allopolyploid. To understand, if one of these mechanisms may have occurred after the initial polyploidization event, artificial neopolyploids would need to be observed across generations.

### Harnessing CWRs to identify traits relevant for crop improvement

For the R-gene example of a ZCR in cultivated sugar beet KWS2320 was observed, that the polyploid *B. corolliflora* and the diploid CWR carry all or at least two out of the five RGA1-5 genes. In wild potato RGA2 confers resistance to the oomycete *Phytophthora infestans* [78, 79]. For *B. corolliflora* and *B. lomatogona*, the gene *RPP8* was found, which confers resistance to *Peronospora parasitica*, which is an oomycete [80, 81]. Multiple genes related to *A. thaliana* R-genes were found, one of which (At3g14460/LRRAC1, found in *B. corolliflora* and *B. macrorhiza*) is associated with defence response to fungal pathogens [82, 83]. Genes that are unique to *B. corolliflora* were e.g. *RPM1*, At5g66890, At5g43730, and the putative late blight resistance protein homolog R1B-19 (*Solanum demissum*). RPM1 confers resistance to some *Pseudomonas syringae* strains [84, 82, 85]. Further, genes associated with stress response to salt and drought, as well as genes associated with the regulation of flowering time, were identified. Genes relevant in response to herbivores include *KTI1* (*B. patula*) and *KTI5* (*B. macrorhiza*), which are involved in the defence response to spider mites (*Tetranychus urticae*) [86]. Spider mites infect a wide range of hosts, one of them being sugar beet [86]. It has been shown that spider mites have a high amount of pesticide resistances, which is why a plants’ natural defence against them is beneficial [85, 87].

Temperature is another important abiotic factor. Freezing temperatures damage sugar beet seedlings; consequently, earlier breeding efforts aimed to develop cold-resistant varieties [88]. Functional gene annotations of the wild beet species examined revealed various genes that could play a role in thermotolerance and cold acclimation. The results suggest that wild beets possess genetic variations that could be significant for adaptation to extreme temperature conditions. However, these findings should be viewed as hypotheses, as sequence and annotation data must be linked with physiological, phenotypic, and functional analyses to clarify whether—and to what extent—these genes contribute to thermotolerance and temperature adaptation.

In summary, the presented method led to the identification of various regions and genes of interest. Even though the model organism *(A) thaliana*, instead of sugar beet itself, had to be used to annotate possible functions, the results show that the genetic variation present in beet wild relatives provides high potential to expand the sugar beet’s gene pool. During the investigation of zero coverage regions (ZCRs), we identified regions derived from *B vulgaris* subsp. *maritima* - the progenitor of sugar beet - and show evidence to support the assumption of sea beet being the progenitor of cultivated sugar beet. Cultivated beets show higher salt tolerance compared to other crops, especially during germination and seed development [89, 90]. The ability to tolerate high salt concentrations is a great advantage for wild sea beets since they are almost exclusively found in coastal regions [91]. In such environments, salt stress represents the most significant abiotic stress. The results of our analysis and the mentioned studies suggest that many of the inherited salt stress-related genes have originated in the sea beet. However, in addition to the stress tolerances from *B. maritima*, *B. corolliflora* harbours several other resistance traits (e.g. frost tolerance) that are of great value for future beet breeding.

## Conclusion

In this study, a sequencing read dataset of sugar beet and CWRs was harnessed to get evidence for the ancestor relationships of a polyploid species. The developed methods to resolve polyploid relationships are based on different concepts and lead to unambiguous results concerning the three tested hypotheses. Therefore, *B. lomatogona* can be excluded as ancestor species of *B. corolliflora.* Further, it can be concluded that *B. macrorhiza* might be the single ancestor of the autotetraploid wild beet *B. corolliflora*. The newly developed approaches used to solve this question can also be applied to other datasets. The generalised trio binning approach seems promising to resolve ancestor relationships and in general phylogenetic relations of closely related species. The investigation of genomic regions not (anymore/yet) present in the cultivated sugar beet genome revealed several genes associated with pathogen resistance and tolerance to abiotic stresses. Both analyses, resolving *B. corolliflora*’s ancestry and identifying genomic regions contributing to stress tolerance, show the potential of the newly generated genome resources of CWRs of sugar beet which areas an essential building block for future investigations of ancestor relationships or other genomic properties.

## Supplementary Information

Additional file 1. Assembly statistics of the new beet genomic resources.

Additional file 2. Chromosomal landmarks along mitotic chromosomes support mostly hypothesis (II) but are not conclusive.

Additional file 3. Results of the synthetic read mapping approach.

Additional file 4: Read datasets used for the assemblies of *B. corolliflora*,* B. lomatogona*,* B. macrorhiza*, and *P. procumbens *and to get insights into the ancestoral relationships of tetraploid *B. corolliflora*.

Additional file 5. Assembly datasets generated in this study and used to get evidence for the ancestor relationships of tetraploid *B. corolliflora*.

Additional file 6. Cutoffs for the assignment of reads to the respective parent candidate(s) assuming that the average k-mer share of a read for both potential parents combined is 0.2.

Additional file 7. Nucleotide sequences of the DNA probes.

Additional file 8. Zero Coverage Regions (ZCRs) in investigated CWRs.

## Data Availability

ONT reads, Illumina reads, and genome assemblies generated for this study were submitted to ENA (PRJEB56520). The sources/IDs are summarised in Additional file 4 and Additional file 5. The structural and functional annotation files for all generated wild beet assemblies are available on ‘PUB-Publications at Bielefeld University’ (https://doi.org/10.4119/unibi/2966932). Relevant scripts for the investigation of the parental relationships of B. corolliflora are available on GitHub (https://github.com/ksielemann/relationships_tetraploid_wild_beet) and have been archived in Zenodo (https://doi.org/10.5281/zenodo.8090593).
