# Supplementary material for: Decoding the autotetraploid origin of the stress-tolerant wild beet Beta corolliflora
Source: BMC Genomics. 2026 Sep 26;27:785. doi: 10.1186/s12864-026-13276-8 (PMC13616124; doi:10.1186/s12864-026-13276-8)
Supplement: Supplementary file 1 [file 12864_2026_13276_MOESM1_ESM.pdf]

Additional file 1: Assembly statistics of the new beet genomic resources. The assemblies of *B. corolliflora*, *B. lomatogona*, and *P. procumbens* are based on long reads, whereas the assembly of *B. macrorhiza* is based on short reads.

Species	***B. corolliflora***	***B. lomatogona***	***P. procumbens***	***B. macrorhiza***
Assembly name	BcorONT v1.0	BlomONT v1.0	PproONT v1.0	Bmrh v1.0
Data type	ONT	ONT	ONT	Illumina
Assembly size [bp]	1963,172,020	1032,079,534	977,011,471	736,230,911
Number of contigs	4,355	1,530	1,542	218,216
N50 [bp]	720,692	1,746,059	1,392,274	7,104
GC content [%]	36.79	36.57	36.53	36.38
Repeat content	1,408,234,706 bp (71.73%)	719,015,079 bp (69.67%)	664,938,575 bp (68.06%)	472,886,165 bp (64.23%)
BUSCOs (n: 2326)	C:95.4% [S:22.5%, D:72.9%], F:0.6%, M:4.0%	C:92.8% [S:58.8%, D:34.0%], F:0.6%, M:6.6%	C:90.1% [S:43.8%, D:46.3%], F:2.6%, M:7.3%	C:68.0% [S:63.9%, D:4.1%], F:12.3%, M:19.7%
