# Supplementary material for: Decoding the autotetraploid origin of the stress-tolerant wild beet Beta corolliflora
Source: BMC Genomics. 2026 Sep 26;27:785. doi: 10.1186/s12864-026-13276-8 (PMC13616124; doi:10.1186/s12864-026-13276-8)
Supplement: Supplementary file 2 [file 12864_2026_13276_MOESM2_ESM.pdf]

Additional file 2: Chromosomal landmarks along mitotic chromosomes support mostly hypothesis (II) but are not conclusive. The number of chromosomes with major, moderate, minor, and no signals was counted for *B. corolliflora*, *B. lomatogona* and *B. macrorhiza* (A). Depending on each hypothesis for *B. corolliflora*'s emergence, the expected counts were deduced (B) and the variance between observed and expected was calculated (C). The average variance between observed and expected for each hypothesis is given in (D).

		Species	Number of chromosomes with signals	18S rDNA	beetSat10/ pRN1	beetSat8/ BlSat1
A	Observation	***B. corolliflora***	strong	**4**	**7**	**5**
		2x = 36	moderate	**0**	**16**	**14**
			weak	**0**	**9**	**17**
			none	**0**	**4**	**0**
		B. lomatogona	strong	2	2	2
		2x = 18	moderate	0	3	4
			weak	0	10	12
			none	0	3	0
		B. macrorhiza	strong	2	3	3
		2x = 18	moderate	0	8	7
			weak	0	5	8
			none	0	2	0
B	Expected *B. corolliflora* counts for each hypothesis	***Hypothesis I***	strong	4	4	4
		2x *B. lomatogona*	moderate	0	6	8
			weak	0	20	24
			none	0	6	0
		Hypothesis II	strong	4	6	6
		2x *B. macrorhiza*	moderate	0	16	14
			weak	0	10	16
			none	0	4	0
		Hypothesis III	strong	4	5	5
		1x *B. lomatogona* &	moderate	0	11	11
		1x *B. macrorhiza*	weak	0	15	20
			none	0	5	0
C	Variance between hypothetic and observed *B. corolliflora* counts	***Hypothesis I***	strong	0%	43%	20%
		2x *B. lomatogona*	moderate	0%	63%	43%
			weak	0%	122%	41%
			none	0%	50%	0%
		Hypothesis II	strong	0%	14%	20%
		2x *B. macrorhiza*	moderate	0%	0%	0%
			weak	0%	11%	6%
			none	0%	0%	0%
		Hypothesis III	strong	0%	29%	0%
		1x *B. lomatogona* &	moderate	0%	31%	21%
		1x *B. macrorhiza*	weak	0%	67%	18%
			none	0%	25%	0%
D	Average variance	***Hypothesis I***		32 % variance to observed signals.		
		Hypothesis II		**4 % variance to observed signals.**		
		Hypothesis III		16 % variance to observed signals.		
		**Conclusion**		**Hypothesis II varies least from the observation. It has most support, but cytogenetic evidence is not conclusive.**		
