# Supplementary material for: Decoding the autotetraploid origin of the stress-tolerant wild beet Beta corolliflora
Source: BMC Genomics. 2026 Sep 26;27:785. doi: 10.1186/s12864-026-13276-8 (PMC13616124; doi:10.1186/s12864-026-13276-8)
Supplement: Supplementary file 3 [file 12864_2026_13276_MOESM3_ESM.pdf]

Additional file 3: Results of the synthetic read mapping approach. A) Composition plot of the subset of synthetic reads of *B. corolliflora* which mapped exclusively to *B. lomatogona* (light orange), exclusively to *B. macrorhiza* (dark orange) and to both species (grey; on the left side; hardly visible due to the small number of synthetic reads mapping to the sequences of both assemblies) for synthetic read lengths of 5 kb, 10 kb, and 20 kb. B) Mapping results (in numbers) for the 5 kb synthetic reads. Most reads (178,984) mapped exclusively to *B. macrorhiza* (Bmrh), less than half as many reads (82,434) mapped exclusively to *B. lomatogona* (Blom), whereas 128,934 reads could not be mapped to any of the potential ancestor species.

A

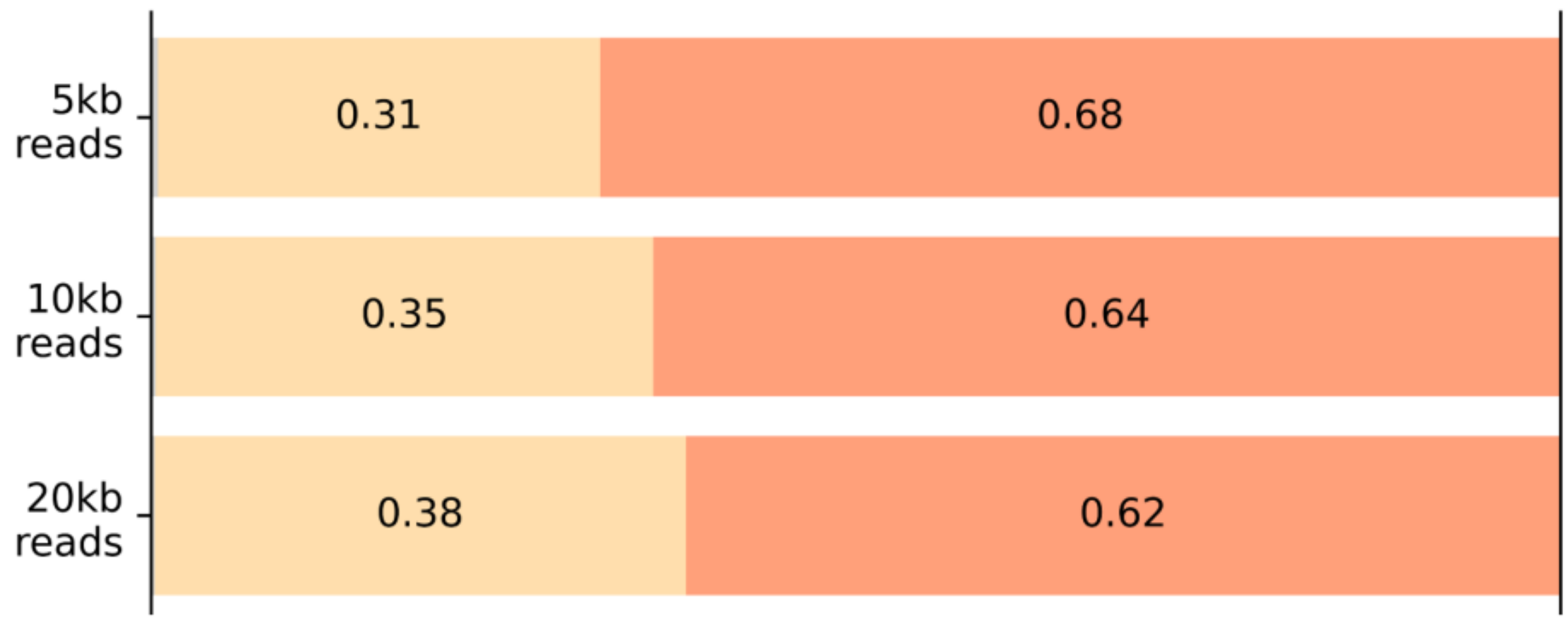

B

5kb reads:

Category	Count
None	128,934
Blom	82,434
Bmrh	178,984
both	1211
