# Supplementary material for: Decoding the autotetraploid origin of the stress-tolerant wild beet Beta corolliflora
Source: BMC Genomics. 2026 Sep 26;27:785. doi: 10.1186/s12864-026-13276-8 (PMC13616124; doi:10.1186/s12864-026-13276-8)
Supplement: Supplementary file 4 [file 12864_2026_13276_MOESM4_ESM.pdf]

Additional file 4: Read datasets used for the assemblies of *B. corolliflora*, *B. lomatogona*, *B. macrorhiza*, and *P. procumbens* and to get insights into the ancestoral relationships of tetraploid *B. corolliflora*.

Name	Technology	SRA/ENA IDs
B. corolliflora	ONT	ERS13530775
	Illumina	ERR6110437, ERR6110438, ERR6110439, ERR6110440, ERR6110441, ERR6110442
B. lomatogona	ONT	ERS13530776
	Illumina	ERR6110445, ERR6110446, ERR6110447, ERR6110448, ERR6110449, ERR6110450
B. macrorhiza	Illumina	ERR6110451, ERR6110452, ERR10496115, ERR10496116, ERR10496117, ERR10496118, ERR10496119, ERR10496120, ERR10496121, ERR10496122, ERR10496123, ERR10496124
P. procumbens	ONT	ERS13530778
	Illumina	ERR10496125, ERR10496126, ERR10496127, ERR10496128, ERR10496129, ERR10496130
B. vulgaris subsp. *vulgaris*	ONT	PRJEB36905
B. napus	ONT	SRR12097594, SRR10383383
B. oleracea	Illumina	ERR2564371, ERR2564372
B. rapa	Illumina	ERR2564158, ERR2564159, ERR6210790
