# Supplementary material for: Decoding the autotetraploid origin of the stress-tolerant wild beet Beta corolliflora
Source: BMC Genomics. 2026 Sep 26;27:785. doi: 10.1186/s12864-026-13276-8 (PMC13616124; doi:10.1186/s12864-026-13276-8)
Supplement: Supplementary file 5 [file 12864_2026_13276_MOESM5_ESM.pdf]

Additional file 5: Assembly datasets generated in this study and used to get evidence for the ancestor relationships of tetraploid *B. corolliflora*.

Name	Technology	Source (ENA/NCBI)	Size [Mb]	Number of contigs	N50 [kb]
B. corolliflora	ONT	ERZ15182113	1963	4355	721
B. lomatogona	ONT	ERZ15182232	1032	1530	1746
B. macrorhiza	Illumina	ERZ15182282	736	218,216	7
B. vulgaris subsp. *vulgaris*	ONT	PRJEB56338 - accession ERZ15181925	573	79	54,420
B. vulgaris subsp. *maritima*	Illumina	GCA_005862445.2 [19]	608	97,415	169
B. patula	Illumina	GCA_005862465.1 [19]	634	78,458	265
P. procumbens	ONT	ERZ15182281	977	1542	1392
B. oleracea	ONT	GCA_900416815.2 [92]	555	129	58,258
B. rapa	ONT	GCA_900412535.3 [92]	444	210	39,218
