# Supplementary material for: Decoding the autotetraploid origin of the stress-tolerant wild beet Beta corolliflora
Source: BMC Genomics. 2026 Sep 26;27:785. doi: 10.1186/s12864-026-13276-8 (PMC13616124; doi:10.1186/s12864-026-13276-8)

Additional file 6: Cutoffs for the assignment of reads to the respective parent candidate(s) assuming that the average *k*-mer share of a read for both potential parents combined is 0.2. The white region in the lower left comprises the reads that do not pass the filtering (below 0.5x the average *k*-mer share = 0.1) and are therefore not considered. Reads located in the region touching the y-axis (orange-red) are classified as belonging to parent B whereas those in the region touching the x-axis (beige) are classified as belonging to parent A. The orange region around the diagonal contains all chimeric reads. The grey regions represent the unclassifiable reads.

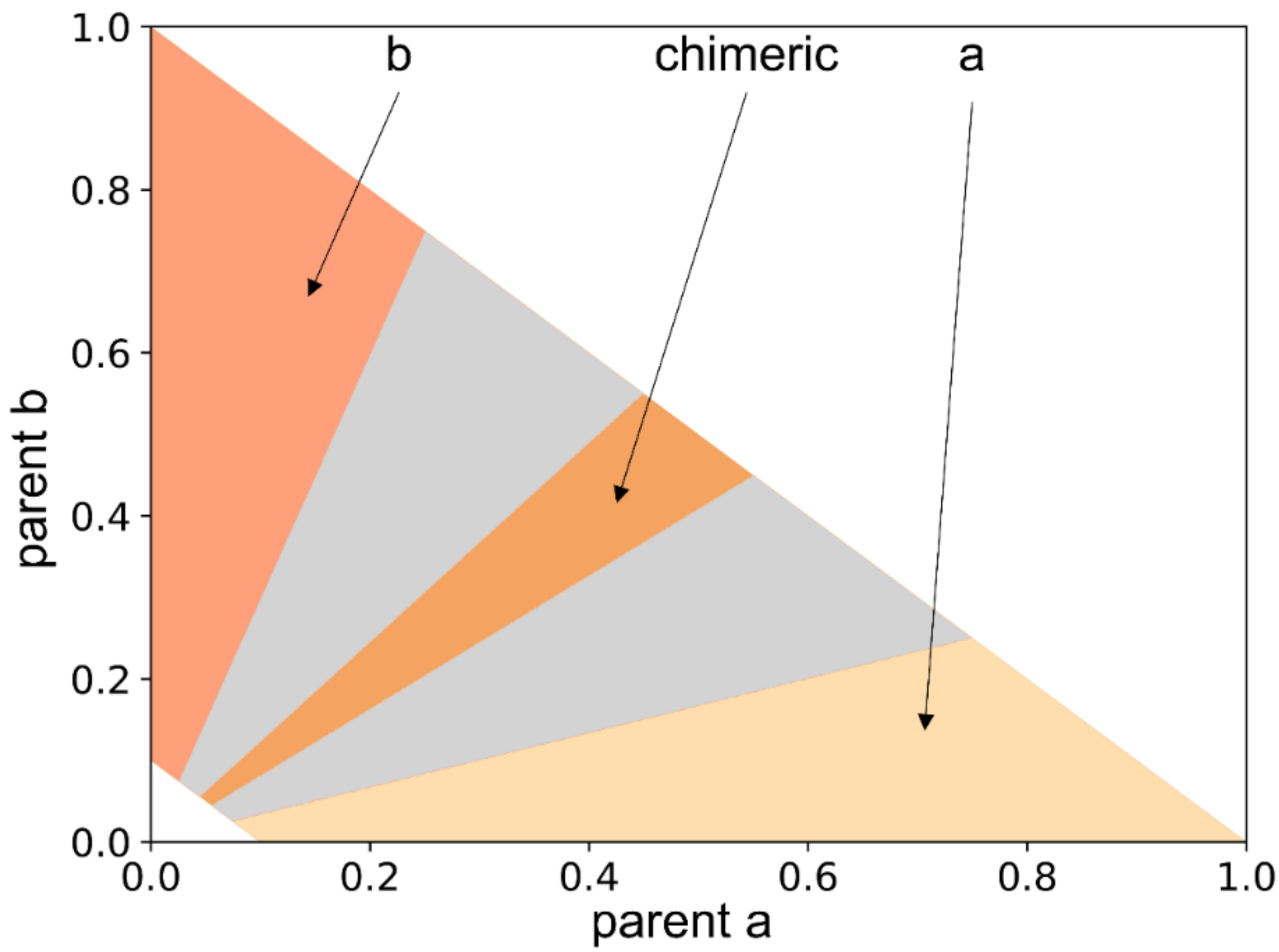

Supplement: Supplementary file 6 [file 12864_2026_13276_MOESM6_ESM.pdf]
