# Supplementary material for: Decoding the autotetraploid origin of the stress-tolerant wild beet Beta corolliflora
Source: BMC Genomics. 2026 Sep 26;27:785. doi: 10.1186/s12864-026-13276-8 (PMC13616124; doi:10.1186/s12864-026-13276-8)
Supplement: Supplementary file 7 [file 12864_2026_13276_MOESM7_ESM.pdf]

Additional file 7: Nucleotide sequences of the DNA probes.

DNA probe	Nucleotide sequence
pRN1	ACTAAAAAGCCCAAATTAACTCCATATCATATGCTTTTTAGTAATTACCAAACATGTTT TGGCCATGACC<br>AAACCTACTAAGTGGAGGATGTTCTCCCAATCACAATCCATTCCCATTACTCATCAAA GCAATCCTATGG<br>GGATTGGACAATCATGCCACTGGGTCCATTAGTCCCAATACCTCTCCAAACCCCATT GACTTCAGTTTTG<br>GGCCCCATTGTCCATCCTTGT
BlSat1	CTAGTTTAAGTTGTAAACACCTCAAACATCATATATATAGTTAGTTTAATGTGATTGTA AGTTGTTTTGATTGAAAATACACAAAATAGGAAAGTTAGAACCAAATATAGGCTAATA TAGGCCATTTTAGGCCATTTCACTTGAAAATGAATGAATACGATTAGGTTGGTGCTAG TTTAAGTTATAAACACCTCAAACATTATA
18S rRNA gene_ Bv_ probe1	GTGCAACAAACCCCGACTTCTGGAAGGGATGCATTTATTAGATAAAAGGTCAACGCG GGCTCTGCCCGTTGCTCTGATGATTCATGATAACTCGACGGATCGCACGGCCTTTG CGCCGGCGACGCATCATTCAAATTTCTGCCCTATCAACTTTCGATGGTAGGATAGTG GCCTACCATGGTGGTGACGGGTGACGGAGAATTAGGGTTCGATTCCGGAGAGGGA GCCTGAGAAACGGCTACCACATCCAAGGAAGGCAGCAGGCGCGCAAATTACCCAAT CCTGACACGGGGAGGTAGTGACAATAAATAACAATACCGGGCTCTTTGAGTCTGGTA ATTGGAATGAGTACAATCTAAATCCCTTAACGAGGATCCATTGGAGGGCAAGTCTGG TGCCAGCAGCCGCGGTAATTCCAGCTCCAATAGCGTATATTTAAGTTGTTGCAGTTA AAAAGCTCGTAGTTGGACCTTGGGGTGGTACGACCGGTCCGCCTTGCGGTGTGCAC CGGTCGTCTTGCCTCTTTCGCCGGCGATGCGCTCCTAGCCTTAA
