# Supplementary material for: Decoding the autotetraploid origin of the stress-tolerant wild beet Beta corolliflora
Source: BMC Genomics. 2026 Sep 26;27:785. doi: 10.1186/s12864-026-13276-8 (PMC13616124; doi:10.1186/s12864-026-13276-8)
Supplement: Supplementary file 8 [file 12864_2026_13276_MOESM8_ESM.pdf]

Additional file 8: Zero Coverage Regions (ZCRs) in investigated CWRs.

About 4.0% (*B. maritima*) to 10.2% (*B. patula*) of the genome sequence assemblies were found to be ZCRs and therefore to be absent and/or not present in sugar beet genotype KWS2320. Absent regions in the cultivated sugar beet KWS2320, but present in the CWRs, were defined as follows. If the region is not present in sugar beet, (almost) no reads of sugar beet should map to the corresponding region in any of the crop wild relatives. Based on this rationale, 'zero coverage regions' (ZCR) were extracted. Reciprocal Best BLAST hits (RBHs) [1] between *A. thaliana* and *Beta* sequences were utilized for the transfer of functional annotations.

**analysis type**	**conserved regions**	**lost regions**	**lost regions**	**lost regions**	**lost regions**	**lost regions**	**lost regions**
species	*B. vulgaris* subsp. *vulgaris*	*P. procum-bens*	*B. patula*	*B. vulgaris* subsp. *maritima*	*B. corolli-flora*	*B. lomato-gona*	*B. macro-rhiza*
genome assembly length [bp]	573,025,584	977,011,471	659,802,716	657,630,391	1,963,172,020	1,032,079,534	736,230,911
number of contigs	79	1542	270,281	463,905	4,355	1530	218,216
mapping rate [%]	86.12	27.99	89.79	92.65	66.36	58.09	60.64
number of variants	9,674,467	3,330,541	12,058,795	11,822,189	26,941,667	22,124,536	20,866,369
variants per position	0.017	0.003	0.018	0.018	0.014	0.021	0.028
mean coverage	108.22	14.38	118.14	126.02	23.25	40.5	56.21
number of passing windows	347,256	313,255	180,685	71,302	294,023	169,689	78,955
number of extended regions	17,608	18,400	17,016	6,562	21,093	11,104	9,680
mean region length [bp]	5,737	4,926	3,962	3,997	4,467	4,691	3,604
max region length [bp]	68,950	188,200	56,350	20,650	130,150	93,850	39,100
number of covered bases	101,005,842 (17.6%)	90,645,550 (9.3%)	67,423,350 (10.2%)	26,225,800 (4.0%)	94,225,650 (4.8%)	52,089,700 (5.0%)	34,891,400 (4.7%)
number of overlapping genes	3,796	970	277	204	1598	1156	2,166
number of genes completely covered	2,038	688	190	125	1220	941	1878
number of RBHs	2,033 (53.6%)	508 (52.4%)	268 (96.8%)	195 (95.6%)	850 (53.2%)	538 (46.5%)	846 (39.1%)
